# Stable carbon and nitrogen isotope dataset from Roman sites in Germania inferior (Xanten, Tongeren, and Valkenburg)

**DOI:** 10.1016/j.dib.2026.112778

**Published:** 2026-04-15

**Authors:** Anna Żmudzka, Maura R.A.L. De Coster, Wouter Vos, Henk van der Velde, Bernd Liesen, Jason E. Laffoon, Lisette M. Kootker

**Affiliations:** a Leiden University, Faculty of Archaeology, Departement of Archaeological Sciences, Einsteinweg 2 2333 CC Leiden, the Netherlands; b Vrije Universiteit Amsterdam, Faculty of Science, Department of Earth Sciences, Geology & Geochemistry cluster, de Boelelaan 1100 1081 HZ Amsterdam, the Netherlands; c Saxion University of Applied Sciences, Handelskade 75, 7417 DH, Deventer, the Netherlands; d Vos Archeo, Van Ewijkweg 41 6861 ZC, Oosterbeek, the Netherlands; e ADC ArcheoProjecten, Nijverheidsweg-Noord 114 3812 PN Amersfoort, the Netherlands; f Vlaams Erfgoed Centrum, Liesdonk 5 2440 Geel, Belgium; g Wissenschaftliche Fundbearbeitung, LVR-Archäologischer Park Xanten / LVR-RömerMuseum, Bahnhofstrasse 46-50, Xanten, Germany; h Vrije Universiteit Amsterdam, Faculty of Humanities, Department of Art and Culture, History, and Antiquity, Antiquity cluster, de Boelelaan 1105 1081 HV Amsterdam, the Netherlands

**Keywords:** Stable isotope analysis, Dietary analysis, Roman period, Archaeology, Northwestern Europe, Roman Limes

## Abstract

This dataset includes stable carbon (*δ*¹³C) and nitrogen (*δ*¹⁵N) isotope measurements from bone and dentine collagen of 38 human and 10 faunal samples dated to the 1st to 4th centuries CE. The material derived from three Roman sites in Germania Inferior that were located along the northwestern frontier of the Roman Empire, the limes, and Via Belgica respectively: Xanten (*Colonia Ulpia Traiana*) in Germany and Valkenburg-Marktveld (*Praetorium Agrippinae*) in the Netherlands, and Tongeren (*Atuatuca Tungrorum*) in Belgium. All analyses were performed using a Thermo DELTA™ Q Isotope Ratio Mass Spectrometer, coupled with a Flash elemental analyser. The isotopic data of the collagen samples are presented in *δ* notation (‰) normalized to the international standard VPDB (Vienna Peedee Belemnite) and atmospheric nitrogen (air) for carbon and nitrogen respectively. The acquired dataset provides a reference for isotopic studies of diet and subsistence in Roman frontier populations and is suitable for reuse in research on dietary reconstruction and for cross-site comparisons. This data article summarises the obtained carbon and nitrogen isotope data, which are deposited in the open access IsoArcH repository (www.isoarch.org) for future studies.

Specifications Table

**Table:** 

Subject	Social Sciences
Specific subject area	Archaeology
Type of data	Raw, Analysed, Table (.csv format), Graph, Figure
Data collection	Human and faunal samples from Roman contexts in Germany, Belgium and the Netherlands were collected for analysis. Carbon and nitrogen stable isotope measurements were obtained by Isotope Ratio Mass Spectrometry. A Thermo DELTA™ Q Isotope Ratio Mass Spectrometer, coupled with a Flash elemental analyser was used to generate the *δ*^13^C and *δ*^15^N data. The data are expressed in *δ* notation (‰), normalised to VPDB for carbon and air for nitrogen.
Data source location	The samples were collected in (WGS84): Xanten **(**Germany**:** 51° 40′ 1.56″ N – 06° 26′ 35.44″ E**)** Tongeren (Belgium: 50° 46′ 49.9″ N – 05° 27′ 53.4″ E) Valkenburg (the Netherlands: 52° 10′ 47″ N – 04° 25′ 54″ E) The data were generated at: Vrije Universiteit Amsterdam, Faculty of Science, Department of Earth Sciences, Stable Isotope Laboratory (VUSIL), Amsterdam, the Netherlands.
Data accessibility	This dataset is deposited in IsoArcH [1,2] (www.isoarch.org) with the following Digital Object Identifier (DOI): https://doi.org/10.48530/isoarch.2026.004. The data are licenced under the BY-SA 4.0 International Creative Commons licence to allow reuse.
Related research article	L.M. Kootker, E. Altena, I. Olalde, A. Veldman, A. Pijpelink, N. Rohland, D. Reich, H. van der Velde, submitted. Beukenbergweg: An unusual burial ground in Early Roman Tongeren, Belgium (International Journal of Osteoarchaeology). M.R.A.L. De Coster, F. Furni, W.K. Vos, J. Oosterbaan, G.R. Davies, L.M. Kootker, submitted. The Roman military community as a melting pot: Biomolecular evidence from the Lower Rhine Limes (Archaeological and Anthropological Sciences).

## 1 Value of the Data

• This dataset presents new stable carbon and nitrogen isotope data from human bone and dentine collagen recovered from Roman sites in Germany, Belgium, and the Netherlands along the north-western frontier of the Roman Empire (Limes/Germania Inferior). It fills a major regional gap and expands the currently limited dataset available for this region.
• The data add to a growing body of carbon and nitrogen analyses of archaeological collagen samples from (pre)historic Europe, thereby supporting chronological investigations into protein sources, trophic levels, and food availability. Moreover, the dataset supports the study of broader processes such as connectivity (“globalisation”) during the 1st–4th centuries CE, providing a valuable complement to archaeological and historical evidence.
• These data are suitable for reuse in future research to expand geographic or temporal comparisons, investigate dietary trends, or to integrate with other isotope systems to address broader paleoenvironmental and archaeological research questions.

## 2 Background

**Fig. 1:**
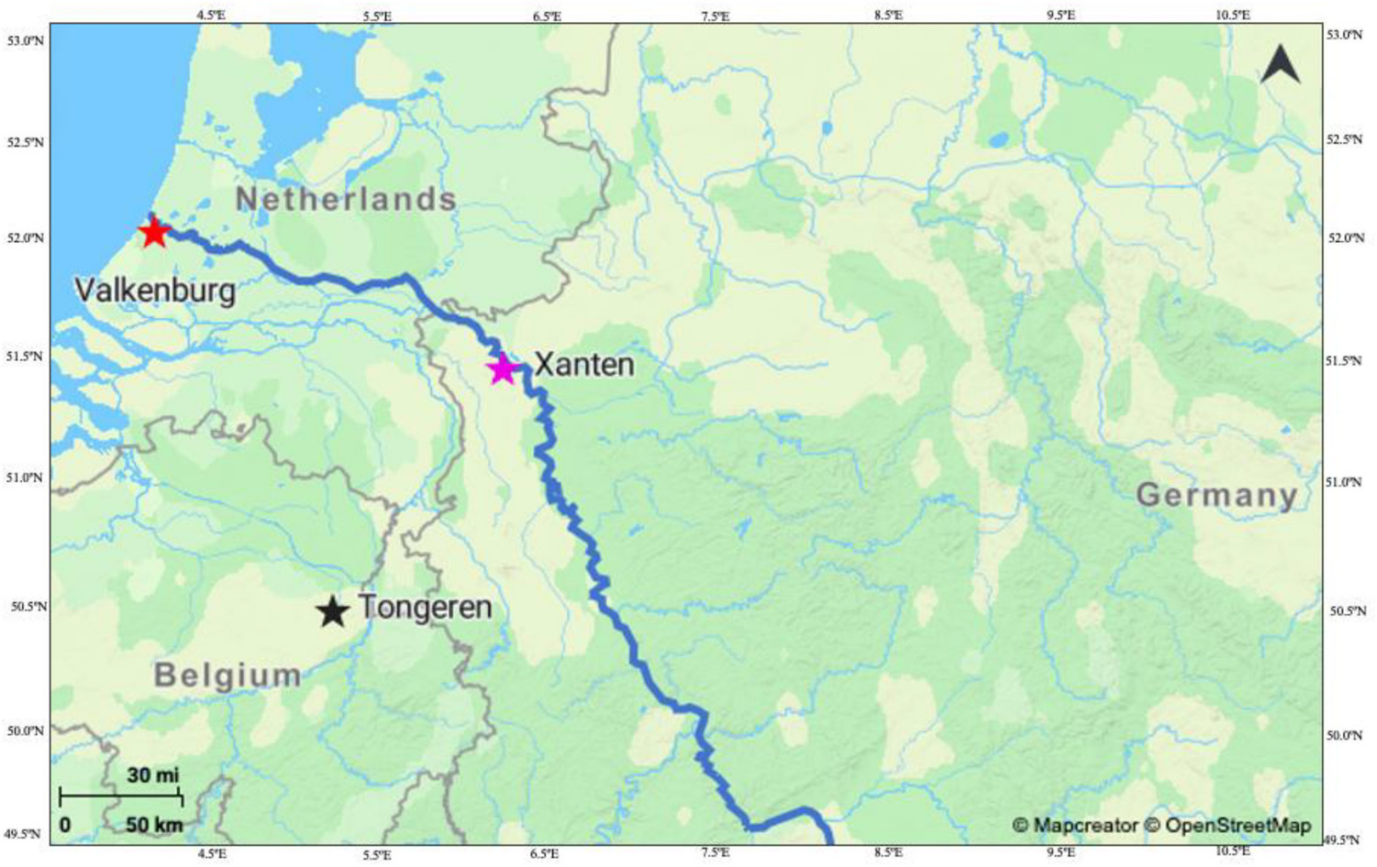
Map showing the sites included in this study. Blue line follows the River Rhine and therefore, a border of Germania Inferior province.

Prior to this study, no comprehensive carbon and nitrogen isotope analyses of bone and dentine collagen had been conducted for the Roman sites of Xanten (Germany), Tongeren (Belgium), and Valkenburg (the Netherlands). Consequently, this dataset was generated to fill a gap in the stable isotope record for the north-western frontier of the Roman Empire. All three sites are situated along the Limes, within the province of Germania Inferior, which broadly followed the course of the Rhine River [3] (Fig. 1). To date, only very limited stable isotope data from this region and period (c. 0–500 CE) have been published or made available through IsoArcH, and existing data are spatially and chronologically fragmented [4,5]. Although modest in size, the present dataset contributes new empirical evidence for this underrepresented region and period and provides a first step towards more systematic future syntheses.

The Roman settlement at Xanten, located on the left bank of the Rhine a few kilometres from the legionary camp *Vetera I* initially served as a port of crucial strategic importance [6]. The settlement received status of *colonia* around 100 CE by the Emperor Traian, becoming *Colonia Ulpia Traiana* [7]. It developed into a major urban centre in Germania Inferior, spanning 73 hectares with an estimated population of 10,000 [8]. Tongeren (*Atuatuca Tungorum*) was founded around 10 BCE in the province of Gallia Belgica. It became one of the new administrative centres and part of the Germania Inferior during the Flavian period [9]. Tongeren was strategically situated on the road between Cologne and Bavay, now known as the *Via Belgica* or *Via Agrippinensis* [[10], [11], [12]]. Valkenburg, located near the Dutch coast on the south bank of Rhine, was an important site along the Roman Limes [13,14]. Crucial structures at this location are, firstly, the short-lived legionary fort, built around 39/40 CE and most likely identifiable as *Praetorium Agrippinae*, and secondly, a smaller military fort (castellum) in line with other castella along the Rhine, such as Alphen aan den Rijn, Woerden, Vleuten de Meern, and possibly also Utrecht. After the fortress disappeared around the middle of the 1st century CE, the site was used as a burial ground for soldiers and civilians; the latter had lived in the vicus in the surrounding area since the Flavian period [15,16].

## 3 Data Description

Within the framework of two projects that focussed on the Limes borderscape and the make-up of the Roman city of Tongeren (see Funding Statement), human and faunal carbon and nitrogen isotope data were generated for 48 samples from Xanten in Germany (*Colonia Ulpia Traiana*); from three locations (Beukenbergweg, Kielestraat, and Zuid-west grafveld) in Tongeren in Belgium (*Atuatuca Tungrorum*); and from Valkenburg in the Netherlands (*Praetorium Agrippinae*, project Marktveld). Initially, a total of 61 bone and dentine samples were collected. Of these, 13 were excluded due to poor collagen preservation or potential contamination, resulting in a final dataset of 48 samples (Table 1).

**Table 1:** Stable carbon (*δ*¹³C) and nitrogen (*δ*¹⁵N) isotope values, and quality indicators for samples analysed in this study. Key: DE = Germany; NL = the Netherlands, BE = Belgium; CUT = *Colonia Ulpia Traiana*; BBW = Beukenbergweg; ZWG = Zuid-west Grafveld; KS = Kielestraat MV = Marktveld.

Find ID	Lab ID	Location	Site	Sample type	Taxon	Collagen yield (%)	%C	δ^13^C vs. VPDB (‰)	%N	δ^15^N vs AIR (‰)	Atomic C:N
50472	X_an_1	Xanten, DE	CUT	Bone	*Sus domesticus*	5.1	39.1	–21.6	14.3	7.1	3.2
49758	X_an_2	Xanten, DE	CUT	Bone	*Bos taurus*	3.5	39	–21.0	14.3	6.8	3.2
51601	X_an_3	Xanten, DE	CUT	Bone	*Mammalia*	9.3	39.6	–21.4	14.6	6.9	3.2
49946	X_an_4	Xanten, DE	CUT	Bone	*Sus domesticus*	10.1	39.8	–21.3	13.9	10.3	3.2
49942	X_an_5	Xanten, DE	CUT	Bone	*Mammalia*	7.7	41	–22.8	15.2	5	3.1
50517	X_an_6	Xanten, DE	CUT	Bone	*Cervus sp*	4.6	38.4	–21.8	14.3	6.5	3.1
50400	X_an_7	Xanten, DE	CUT	Bone	*Cervus sp*	0.9	33.7	–22.1	12.3	7.8	3.2
50414	X_an_8	Xanten, DE	CUT	Bone	*Cervus* sp.	4.4	37.8	–21.1	13.9	8.8	3.2
51197	X_an_9	Xanten, DE	CUT	Bone	*Bos taurus*	-	-	-	-		-
49930	X_an_10	Xanten, DE	CUT	Bone	*Sus domesticus*	13.3	39.6	–22.1	14.7	7.1	3.2
50589	X_an_11	Xanten, DE	CUT	Bone	*Aves*	9.3	40.6	–20.2	15	12.1	3.2
5948b	X_SK1	Xanten, DE	CUT	Bone	*Homo sapiens*	4.7	-	-	-	-	-
	X_SK1d	Xanten, DE	CUT	Dentine	*Homo. sapiens*	7.6	39.7	–19.8	14.1	11.5	3.3
5846	X_SK2	Xanten, DE	CUT	Bone	*Homo sapiens*	4.8	37.1	–19.4	13.7	10.6	3.2
	X_SK2d	Xanten, DE	CUT	Dentine	*Homo sapiens*	5.1	38	–19.3	13.9	10.2	3.2
5942	X_SK3	Xanten, DE	CUT	Bone	*Homo. sapiens*	16.6	38.7	–20.3	14.2	10.2	3.2
	X_SK3d	Xanten, DE	CUT	Dentine	*Homo sapiens*	14.4	38.1	–19.9	14.1	11.4	3.2
7022	X_SK4	Xanten, DE	CUT	Bone	*Homo sapiens*	4.2	36.5	–19.2	13.7	11.4	3.1
	X_SK4d	Xanten, DE	CUT	Dentine	*Homo sapiens*	13.3	39.9	–18.3	14.7	10.4	3.2
5903	X_SK5	Xanten, DE	CUT	Bone	*Homo sapiens*	14.6	40.2	–19.5	15	11.9	3.1
	X_SK5d	Xanten, DE	CUT	Dentine	*Homo sapiens*	9.1	39.5	–19.7	14.5	11.3	3.2
5820	X_SK6	Xanten, DE	CUT	Bone	*Homo sapiens*	5.6	38	–19.8	14.1	11.3	3.1
	X_SK6d	Xanten, DE	CUT	Dentine	*Homo sapiens*	9.4	37.9	–19.5	14.2	12.7	3.1
24149/48	X_SK7	Xanten, DE	CUT	Bone	*Homo sapiens*	2.9	38.2	–19.1	14.3	9.2	3.1
	X_SK7d	Xanten, DE	CUT	Dentine	*Homo sapiens*	7.1	-	-	-	-	-
6989	X_SK8d	Xanten, DE	CUT	Dentine	*Homo sapiens*	9.4	37.9	–18.8	14.1	11	3.1
24143	X_SK9d	Xanten, DE	CUT	Dentine	*Homo sapiens*	17.3	37.4	–18.6	13.8	9.7	3.2
7313	X_SK10d	Xanten, DE	CUT	Dentine	*Homo sapiens*	21.2	38.7	–18.5	14.4	12.4	3.1
7473	X_SK11d	Xanten, DE	CUT	Dentine	*Homo sapiens*	21.5	37.2	–19.5	13.8	11.5	3.2
8046	X_SK12d	Xanten, DE	CUT	Dentine	*Homo sapiens*	21.7	-	-	-	-	-
5861	X_SK13d	Xanten, DE	CUT	Dentine	*Homo sapiens*	21.5	38.3	–19.2	14.3	8.8	3.1
5836	X_SK14d	Xanten, DE	CUT	Dentine	*Homo sapiens*	17.3	38.3	–19.8	14.4	10.4	3.1
5845	X_SK15	Xanten, DE	CUT	Bone	*Homo sapiens*	1.5	-	-	-	-	-
2856	X_SK16d	Xanten, DE	CUT	Dentine	*Homo sapiens*	13.1	39.4	–18.8	14.2	11	3.3
24148/49	X_SK17d	Xanten, DE	CUT	Dentine	*Homo sapiens*	21.4	38.3	–19.1	14.2	9.3	3.2
900	T_SK1	Tongeren, BE	BBW	Bone	*Homo sapiens*	5.6	40.7	–19.3	14.5	10.8	3.3
1081	T_SK2	Tongeren, BE	BBW	Bone	*Homo sapiens*	3.5	40.7	–20.1	14.5	10.8	3.3
884	T_SK3	Tongeren, BE	BBW	Bone	*Homo sapiens*	8.3	35.1	–19.5	12.2	11.3	3.4
1078	T_SK4	Tongeren, BE	BBW	Bone	*Homo sapiens*	8.8	39.3	–19.7	13.8	11.8	3.3
1314	T_SK5	Tongeren, BE	BBW	Bone	*Homo sapiens*	9.1	43.4	–19.1	15.5	11	3.3
1622	T_SK6	Tongeren, BE	BBW	Bone	*Homo sapiens*	8.1	39.6	–18.5	10.3	10	3.3
1746	T_SK7	Tongeren, BE	BBW	Bone	*Homo sapiens*	19.1	42.1	–18.9	14.9	11.5	3.3
1082	T_SK8	Tongeren, BE	BBW	Bone	*Homo sapiens*	6.2	45.3	–19.4	16.2	9.9	3.3
G28	T_SK9	Tongeren, BE	ZWG	Bone	*Homo sapiens*	6.1	35.8	–19.5	12.3	10.3	3.4
G99	T_SK10	Tongeren, BE	ZWG	Bone	*Homo sapiens*	5.6	43	–19.7	15.4	9.1	3.3
G119	T_SK11	Tongeren, BE	ZWG	Bone	*Homo sapiens*	3.1	40.1	–19.5	14.2	10.2	3.3
G92B	T_SK12	Tongeren, BE	KS	Bone	*Homo sapiens*	6.4	41.9	–19.3	15	9.6	3.3
G92C	T_SK13	Tongeren, BE	KS	Bone	*Homo sapiens*	2.8	40.9	–20.0	14.4	8.9	3.3
G7/v375	T_SK14	Tongeren, BE	KS	Bone	*Homo sapiens*	11.2	42.5	–20.2	15.1	9.5	3.3
G3/V464	T_SK15	Tongeren, BE	KS	Bone	*Homo sapiens*	7.2	40.6	–19.9	14.3	11	3.3
G3/459	T_SK16	Tongeren, BE	KS	Bone	*Homo sapiens*	6.9	40.5	–21.9	14.4	8.7	3.3
G92A	T_SK17	Tongeren, BE	KS	Bone	*Homo sapiens*	10.1	35.2	–19.9	12.1	9.2	3.4
I1	V_SK2	Valkenburg, ZH, NL	MV	Bone	*Homo sapiens*	6.5	37.8	–19.9	13.4	10.3	3.3

A total of 35 samples were collected from Xanten, comprising of 11 faunal remains (mammal), and 24 human remains samples, including both human bones (*n* = 8) and teeth (*n* = 16). These human remains represent 17 individuals, as some teeth and bones belonged to the same individuals.

Samples from Tongeren and Valkenburg consisted exclusively of human bone. Seventeen samples were obtained from Tongeren, distributed across three locations: Beukenbergweg (*n* = 8), Zuid-west Grafveld (*n* = 3), and Kielestraat (*n* = 6). Nine samples were collected from Valkenburg-Marktveld, however only one sample was suitable for isotopic analysis, as the remaining eight were excluded due to poor preservation or insufficient collagen yield.

Bone collagen was primarily extracted from human ribs, though six crania and three long bones from Tongeren were also sampled. In the case of successfully extracted bone collagen from Valkenburg, a long bone was used. Long bones of the faunal assemblage were selected, as they provide the most reliable basis for species-level identification of skeletal elements. The average stable carbon and nitrogen isotope values per site, together with a summary of the associated descriptive statistics, are presented in Table 2.

**Table 2:** Descriptive statistics of stable carbon and nitrogen isotope values from the studied sites. Valkenburg site not included, as only one sample was suitable for analysis.

			*δ*^13^C_VPDB_ (‰)					*δ*^15^N_air_ (‰)				
Location	Site	Material	Mean	SD	Min	Max	range	Mean	SD	Min	Max	range
**Xanten**	Colonia Ulpia Traiana	Faunal bone collagen	–21.5	0.7	–22.8	–20.2	2.6	7.8	2.1	5	12.1	7.1
		Human bone collagen	–19.6	0.4	–20.3	–19.1	1.2	10.8	1.0	9.2	11.9	1.7
		Human dentine collagen	–19.2	0.5	–19.9	–18.3	1.6	10.8	1.1	8.8	12.7	3.9
**Tongeren**	All sites	Human bone collagen	–19.7	0.7	–21.9	–18.5	3.4	10.2	1.0	8.7	11.8	3.1
	Beukenbergweg	Human bone collagen	–19.3	0.5	–20.1	–18.5	1.6	10.9	0.7	9.9	11.8	1.9
	ZW Grafveld	Human bone collagen	–19.6	0.1	–19.7	–19.5	0.2	9.9	0.7	9.1	10.3	1.2
	Kielestraat	Human bone collagen	–20.2	0.9	–21.9	–19.3	2.6	9.5	0.8	8.7	11.0	2.3

**Fig. 2:**
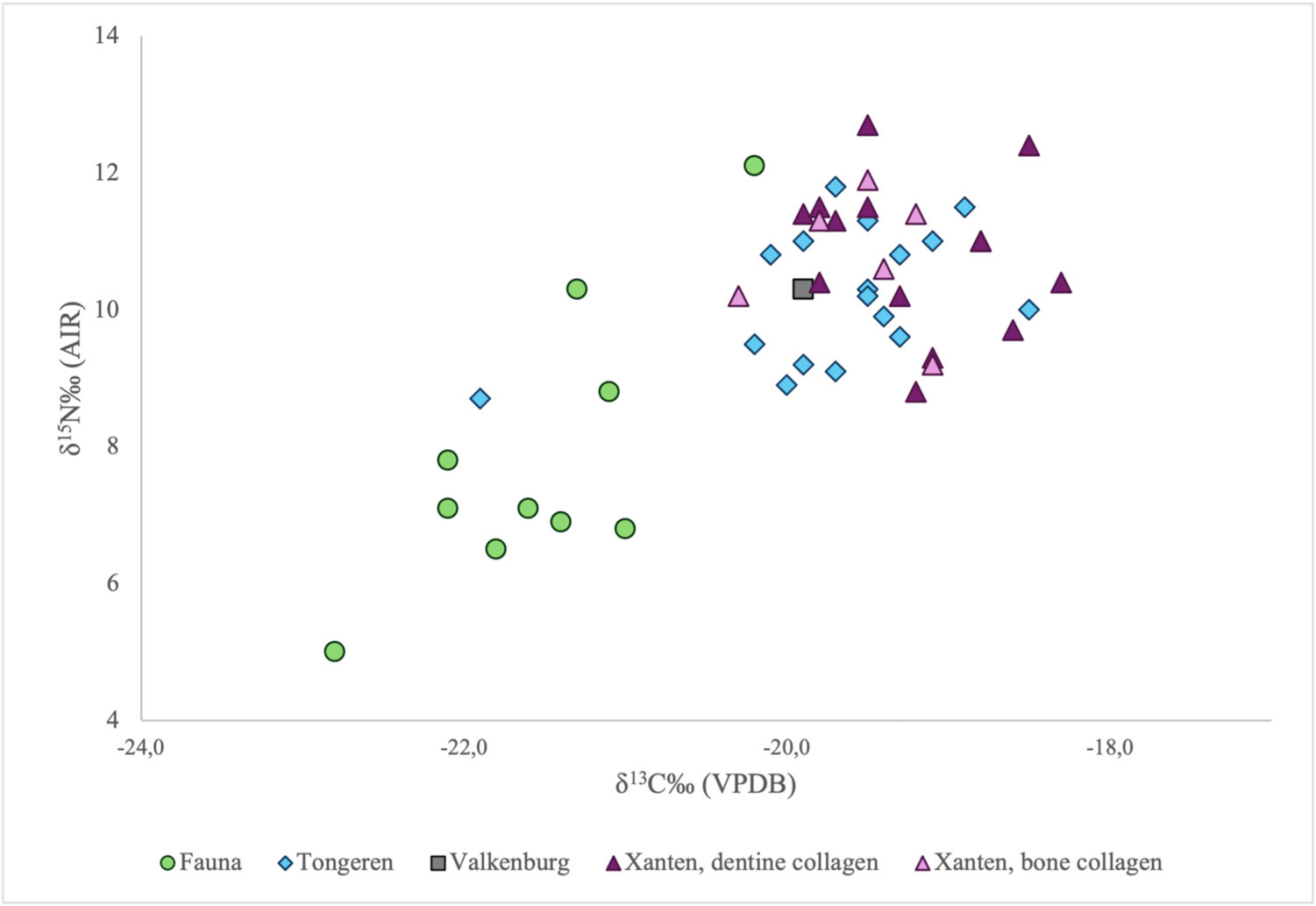
Stable carbon (*δ*^13^C) and nitrogen (*δ*^15^N) isotope values for faunal remains from Xanten and all human isotopic values from this study.

**Fig. 3:**
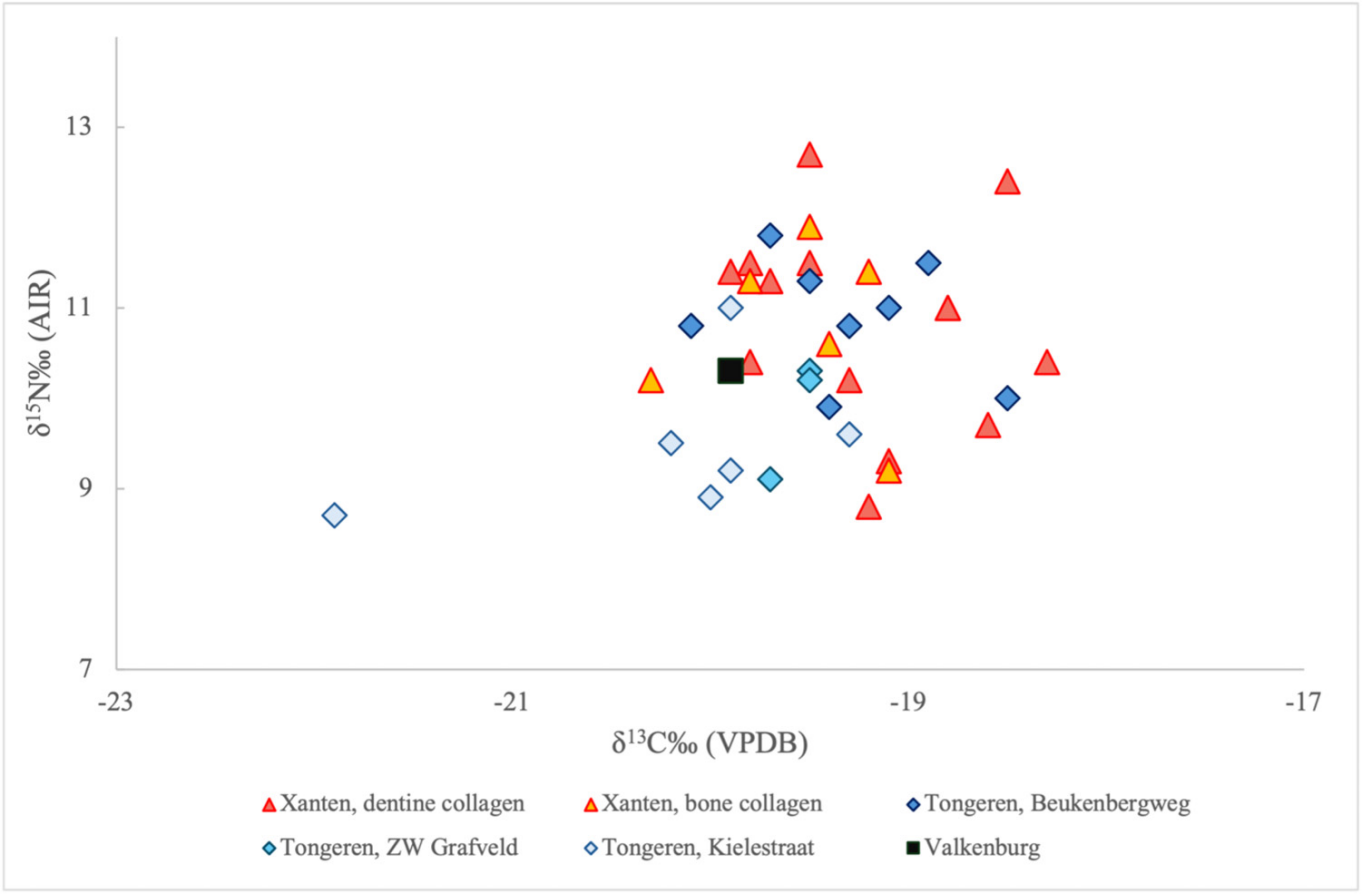
Stable carbon (*δ*^13^C) and nitrogen (*δ*^15^N) isotope values of samples from all sites, grouped by tissue type for Xanten and by spatial subdivision for Tongeren.

**Fig. 4:**
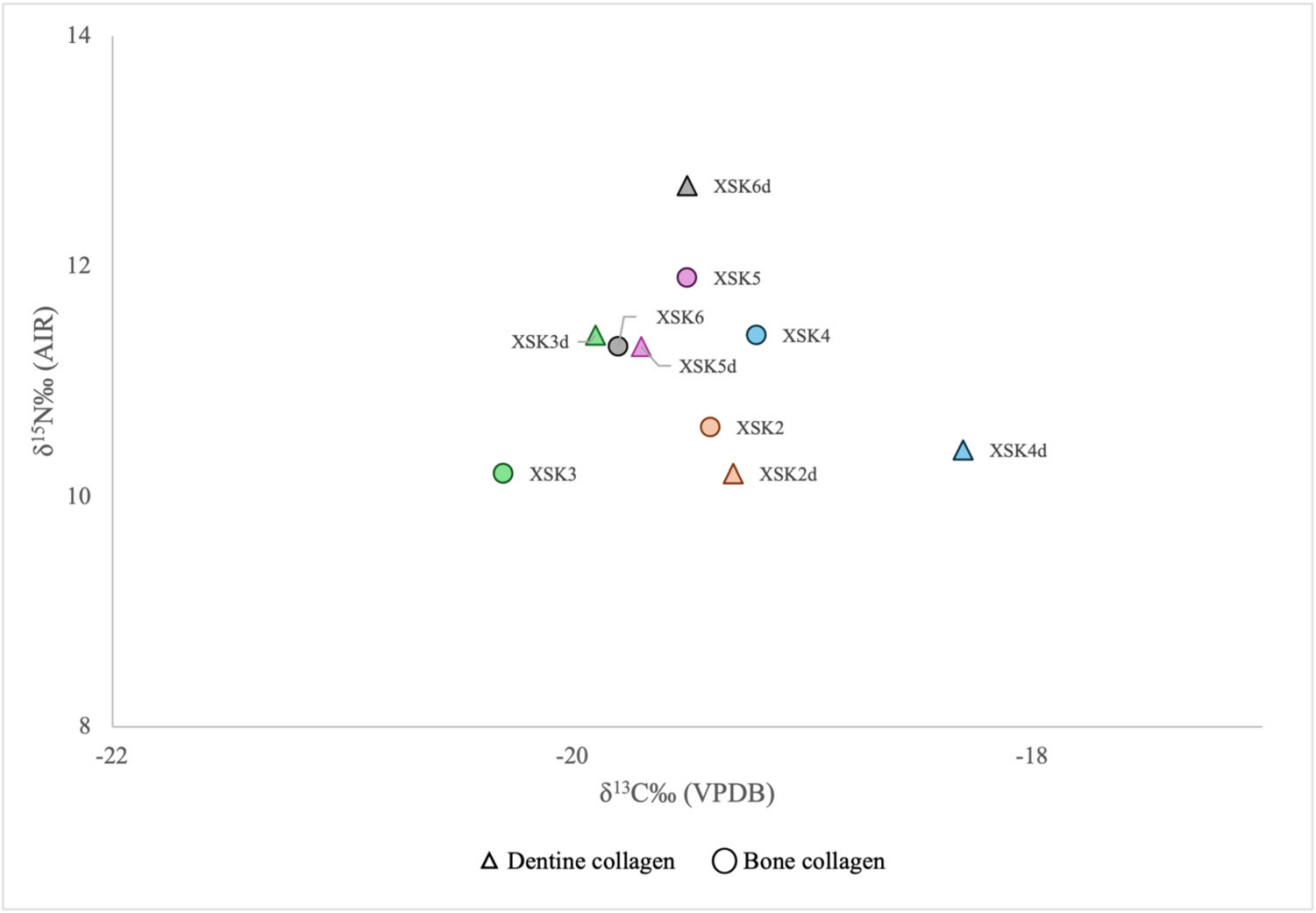
Stable carbon (*δ*^13^C) and nitrogen (*δ*^15^N) isotope values of dentine and bone collagen samples derived from the same individuals at the Xanten site.

The database includes stable isotope measurements from three archaeological sites incorporating both bone and dentine collagen samples. Data are organised by site and, where applicable, by spatial subdivision within each site. A limited number of faunal bone collagen *δ*^13^C and *δ*^15^N isotope values (*n* = 11) are included in the dataset to provide a comparative reference for the human isotope measurements generated in this project. Fig. 2 presents a visual overview of the *δ* ^13^C and *δ* ^15^N values recorded for the faunal and human bone collagen samples included in the dataset. Fig. 3 presents the *δ* ^13^C and *δ* ^15^N values for all samples included in the dataset. For Xanten, the dataset includes paired bone and dentine collagen samples derived from the same individual. Fig. 4 presents the *δ* ^13^C and *δ* ^15^N values of these paired samples.

## 4 Experimental Design, Materials and Methods

**Fig. 5:**
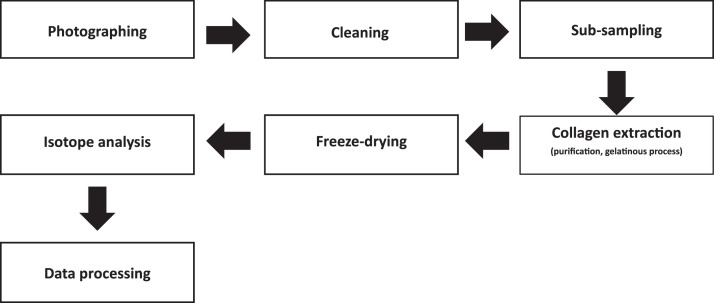
Simplified workflow of data acquisition and analysis steps.

For the analysis, small, already fragmented rib specimens were selected from the human assemblage. In cases where complete or large rib fragments or long bones were present, a small sample (c. 2 cm³) was taken, either by manual sampling using a small metal saw or with a Dremel equipped with a small cutting disc. The disc was cleaned with diluted hydrochloric acid (10% HCl) between samples to prevent cross-contamination. Each sample was photographed prior to the laboratory processes. Fig. 5 illustrates the simplified workflow of the data acquisition procedure. Initially, the samples were cleaned ultrasonically for circa 15–20 min in demineralised water to remove surface contaminants. This step was repeated until the water in the beaker remained clear. Subsequently, the samples were dried in a fume hood for approximately 48 h until completely dry. Next, approximately 200–300 mg of dry bone was subsampled. The only exception concerned the faunal samples for which subsample weights ranged from 500 to 1000 mg. Collagen extraction was performed following the standard Leiden University Chemistry Lab protocol based on [17]. The subsamples retained its original identification number (find number) and were placed into pre-cleaned, labelled tubes. Glass tubes were used for bone subsamples that required filtration in subsequent steps, whereas plastic tubes were used for dentine collagen subsamples, for which filtration was not necessary.

The samples were then demineralised to remove the inorganic components from the bones. In this step, 10 ml of 0.6 M hydrochloric acid (HCl) was added to each tube using a pipette or dispenser. Each tube was securely sealed using fitted caps, gently shaken, and stored in a refrigerator at approximately 4 °C for 48 h. After this period, the acid was removed and replaced with fresh acid. This procedure was repeated until the bones became soft and no further reaction between the acid and the material was observed. The duration of this step varied depending on the sample and typically lasted 6 to 8 days, but this was occasionally extended up to 12 days.

Once demineralisation was completed, the acid was removed, and the samples were rinsed with Milli-Q^Ⓡ^ water (Millipore, Bedford, MA, USA; >18 M Ω purity) to eliminate any remaining acid residues. The samples were then centrifuged at 2000 rpm for 1 min, the rinsing step was repeated three times. In the subsequent purification step, 10 ml of 0.125 M alkaline sodium hydroxide (NaOH) solution was added to each subsample. The tubes were left for approximately 20 h at room temperature in a fume hood to ensure the removal of humic acid. After this period, the alkaline solution was decanted, and the samples were rinsed with Milli-Q^Ⓡ^ water and centrifuged at 2000 rpm for 1 min. This step was also repeated three times.

The next step involved gelatinisation, in which 9 ml of 0.001 M HCl (pH = 3) was added to each tube. The tubes were then placed in an oven at 80 °C for approximately 48 to 72 h. As this step is sample-dependent, the duration was extended for some samples. After the samples were cooled to room temperature, they were vortexed and filtered using 60–90 µm mesh Ezee-filter™ separators (Elkay^Ⓡ^ Laboratory Products). The filtrates were then sealed with Parafilm^Ⓡ^ and frozen overnight. Once frozen, the samples were freeze-dried for 48 h. The resulting collagen was then weighed on a laboratory balance to calculate collagen yield using the following equation:

$$Collagen\;yield\;\%=\frac{weight\;collagen}{weight\;of\;dry\;bone\;\left(subsample\right)}\times 100$$

The tubes were sealed with their caps and stored in a dark, dry environment until further processing.

The samples were transported to the Stable Isotope Laboratory of the Vrije Universiteit Amsterdam (VUSIL). Here, approximately 0.5 mg ± 10% of purified collagen was weighted into 6 × 4 mm tin capsules using a nanoscale, folded, and placed in corresponding trays. In addition, international standard samples (USGS40, USGS41, USGS42), were sampled alongside the samples to calibrate the instrument and monitor the analytical precision and accuracy. Isotopic analysis was performed at the same laboratory using a Thermo DELTA™ Q Isotope Ratio Mass Spectrometer, coupled with a Flash elemental analyser. During this analysis, the aliquots were combusted at high temperature to convert them from solid to gas. The resulting isotope ratios are expressed in *δ* notation in per mille (‰), and normalised to VPDB for carbon and air for nitrogen.

Data quality was monitored by evaluating the atomic ratio of carbon-to-nitrogen (C/N * 14/12) of each sample, an established indicator of collagen preservation and suitability for further interpretation. Acceptable C/N ratios generally fall between 2.9 and 3.6; the samples exhibiting values outside this range were excluded from further interpretation [18]. Furthermore, collagen integrity and degradation were assessed by determining the %C and %N. For archaeological collagen, the %C values greater than 13% and %N greater than 4.5% are typically indicative of well-preserved collagen [19]. The %C and %N were calculated using the following equations:

$$\%C=\frac{mass\;of\;carbon\;in\;aliquot\;sample}{mass\;of\;aliquot}\times 100$$

$$\;\%N\;=\frac{mass\;of\;nitrogen\;in\;aliquot\;sample\;}{mass\;of\;aliquot}\times 100$$

## Limitations

The dataset is limited in several ways related to sample size and spatial coverage. Of the 61 bone and dentine samples originally collected, 13 were excluded due to poor collagen preservation or potential contamination, resulting in a final dataset of 48 samples. The geographic scope is restricted to three Roman-period sites (Xanten, Tongeren, Valkenburg) in northwestern Europe, representing only a limited portion of the region. Additionally, the number of faunal samples is relatively small (*n* = 11), which constrains the resolution of the local isotopic baselines. These factors should be considered when using the dataset, particularly for studies requiring larger sample sizes, wider geographic representation, or more robust faunal reference frameworks.

## Ethics Statement

The authors have read and comply with the ethical requirements for publication in Data in Brief and confirm that this work does not involve modern human subjects, animal experiments, or data collected from social media platforms.

## Credit Author Statement

**Anna Żmudzka**: Methodology, Data curation, Formal analysis, Visualisation, Writing - Original Draft. **Maura R.A.L De Coster**: Conceptualization, Project administration, Writing - Review & Editing. **Wouter Vos**: Resources, Project administration, Writing - Review & Editing. **Henk van der Velde**: Resources, Funding acquisition, Writing - Review & Editing. **Bernd Liesen**: Resources, Writing - Review & Editing. **Jason E. Laffoon**: Resources, Supervision, Writing - Review & Editing. **Lisette M. Kootker**: Conceptualisation, Resources, Supervision, Funding acquisition, Writing - Review & Editing.

## Data Availability

IsoArchStable carbon and nitrogen isotope dataset from Roman sites in Germania Inferior (Xanten, Tongeren, and Valkenburg). (Original data).
